# A Determination of Potential α-Glucosidase Inhibitors from Azuki Beans (*Vigna angularis*)

**DOI:** 10.3390/ijms12106445

**Published:** 2011-09-28

**Authors:** Yang Yao, Xuzhen Cheng, Lixia Wang, Suhua Wang, Guixing Ren

**Affiliations:** Institute of Crop Science, Chinese Academy of Agricultural Sciences, South Xueyuan Road, Haidian District No.80, Beijing 100081, China; E-Mails: yaoyang@caas.net.cn (Y.Y.); chengxz@caas.net.cn (X.C.);wanglx@caas.net.cn (L.W.); wangsh@caas.net.cn (S.W.);

**Keywords:** azuki beans, α-glucosidase inhibitory, vitexin, isovitexin

## Abstract

A 70% ethanol extract from azuki beans (*Vigna angularis*) was extracted further with CH_2_Cl_2_, EtOAc and n-BuOH to afford four fractions: CH_2_Cl_2_-soluble, EtOAc-soluble, *n*-BuOH-soluble and residual extract fractions. The EtOAc-soluble fractions showed the highest α-glucosidase inhibitory activity. Two pure flavonoid compounds, vitexin and isovitexin, were isolated (using the enzyme assay-guide fractionation method) from the EtOAc-soluble fractions. We further evaluated the interaction between the flavonoid compounds and α-glucosidase by fluorescence spectroscopy. Vitexin and isovitexin showed high inhibitory activities, with IC_50_ values of 0.4 mg·mL^−1^ and 4.8 mg·mL^−1^, respectively. This is the first study of the active compositions of azuki beans against α-glucosidase.

## 1. Introduction

Interest in glucosidase inhibitors is growing because of its implications for the management of diabetes mellitus (DM). DM is a serious metabolic disorder that affects approximately 4% of the population worldwide and is expected to increase to affect 5.4% by 2025 [1]. Acting as a key enzyme for carbohydrate digestion, intestinal α-glucosidase is one of the glucosidases located at the epithelium of the small intestine. α-glucosidase has been recognized as a therapeutic target for the modulation of postprandial hyperglycemia, which is the earliest metabolic abnormality to occur in type 2 diabetes mellitus [2,3]. The inhibition on intestinal α-glucosidases would delay the digestion and absorption of carbohydrates and consequently suppress the postprandial hyperglycemia [4].

Azuki beans have been a subject of extensive investigation due to their biological activities. In the past, they have been recommended as suitable foods for diabetic patients due to their high fiber and protein contents [5]. Recently, they have also been reported to contain considerable quantities of bioactive phytochemicals including phenolic compounds [6], which may offer extra benefits for the amelioration of diabetes. Itoh *et al*. [7] reported that azuki beans possess inhibition activity against α-glucosidase in streptozotocin (STZ)-induced diabetic rats. However, the studies on anti-diabetic effects were focused on the activity of the extract; the active components of the extract were not ascertained. The present study was therefore carried out to isolate and identify the active compositions of azuki beans by enzyme assay-guided fractionation.

## 2. Results and Discussion

### 2.1. Isolation of Active Compounds and Structural Determination

**Figure 1:**
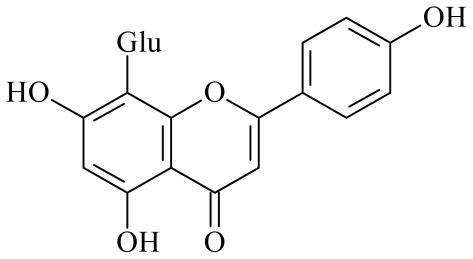
Chemical structures of vitexin.

**Figure 2:**
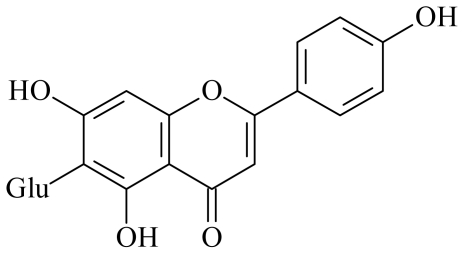
Chemical structures of isovitexin.

Itoh *et al*. [7] investigated the antidiabetic effects of azuki beans on streptozotocin (STZ)-induced diabetic rats. We also observed that azuki beans showed the highest α-glucosidase inhibition ability among sixteen legumes (data not shown). However, the active components of the extract were not ascertained. In this study, two pure compounds were separated from the EtOAc-soluble fraction by the method mentioned above; they were identified as vitexin and isovitexin (structures are shown in Figures 1 and 2), by comparison of their spectral data with those in the literature [8,9].

*Vitexin* (**1**): yellow powder. ^1^H NMR (500 MHz, DMSO-*d*6) d: 13.15 (1H, s, OH-5), 8.01 (2H, d, *J =* 8.7 Hz, H-2′, 6′), 6.86 (2H, d, *J =* 8.7 Hz, H-3′, 5′), 6.76 (1H, s, H-3), 6.23 (1H, s, H-6), 4.69 (1H, d, *J =* 9.8 Hz, H-1′ of glu), 3.85–3.22 (6H, m, glucosyl H). ^13^C NMR (DMSO-*d* 6) d: 182.0 (C-4), 164.8 (C-2), 162.7 (C-7) 161.3 (C-4′), 160.4 (C-5),156.09 (C-9), 128.5 (C-2′, 6′), 121.2 (C-1′), 115.8 (C-3′, 5′), 104.7 (C-10), 104.1 (C-8),102.4 (C-3), 98.2 (C-6). Positive ESI-MS: *m*/*z* 433 [M + H]^+^.

Isovitexin (**2**): yellow powder. ^1^H NMR (500 MHz, DMSO-*d*6) d: 13.55 (1H, s, OH-5), 7.93 (2H, d, *J =* 8.8 Hz, H-2′, 6′), 6.93 (2H, d, *J =* 8.8 Hz, H-3′, 5′), 6.78 (1H, s, H-3), 6.51 (1H, s, H-8), 4.58 (1H, d, *J =* 9.8 Hz, H-1 of glu), 4.03–3.11 (6H, m, glucosyl H). ^13^C NMR (DMSO *d*6) d: 181.9 (C-4), 163.5 (C-2), 163.3 (C-7), 161.6 (C-4′), 160.6 (C-5), 158.2 (C-9), 128.4 (C-2′, 6′), 121.1 (C-1′), 116.06 (C-3′, 5′), 108.9 (C-6), 103.4 (C-10), 102.8 (C-3), 93.64 (C-8). Positive ESI-MS: *m*/*z* 433 [M + H]^+^.

### 2.2. Alpha-Glucosidase Inhibition Activities

To determine the α-glycosidase inhibition ability *in vitro*, we calculated the IC_50_ values (Table 1). The EtOAc-soluble fraction had the highest α-glucosidase inhibitory activity of the four partition parts. Vitexin was the most active (IC_50_ of 0.4 mg·mL^−1^), followed by isovitexin (IC_50_ of 4.8 mg·mL^−1^).

### 2.3. Fluorescence Spectra

Fluorescence quenching can be divided into two types: dynamic quenching and static quenching. Dynamic quenching stems from the collision between two fluorescent luminophors, while static quenching arises from the formation of a new nonfluorescent complex that forms between the fluorescent luminophors and quencher [10]. Dynamic quenching follows the Stern–Volmer equation [11]:

(1) $$F_{0}/F=1+K_{\text{sv}}[Q]=1+K_{\text{q}}\tau_{0}[Q]$$

where *F*_0_ and *F* are the fluorescence intensities of the fore-and-aft interaction between α-glucosidase and flavonoid, [*Q*] is the concentration of quencher and flavonoid, and *τ*_0_ is the average life of the fluorescent substance without the quencher, valued at approximately 10^−8^ s. *K*_sv_ and *K*_q_ are the dynamic quenching constant and rate constant in the process of double molecule quenching [12].

**Figure 3:**
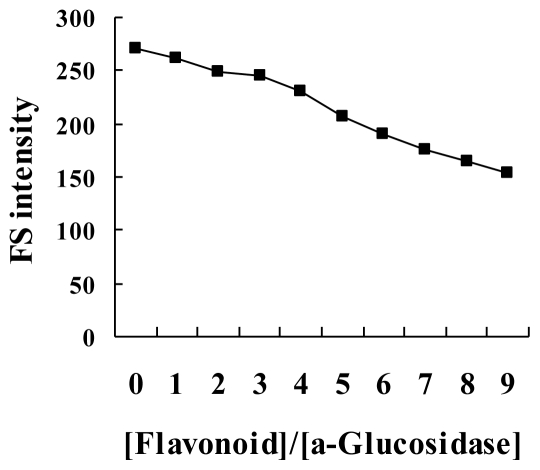
The effect of vitexin on fluorescence spectrum of α-glucosidase after they were added to the enzyme solution.

**Figure 4:**
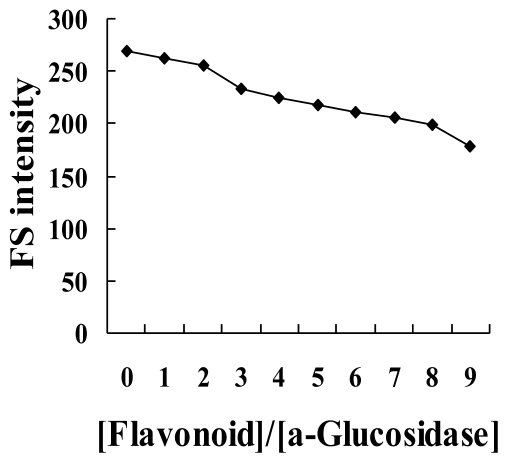
The effect of isovitexin on fluorescence spectrum of α-glucosidase after they were added to the enzyme solution.

The quenching fluorescence spectra of α-glucosidase by flavonoids were recorded at 25 and 37 °C (Figures 3 and 4). The values of *K*_sv_ and *K*_q_ were obtained with the Stern-Volmer equation from plots of linear equations obtained by *F*_0_/*F vs.* [*Q*]. The values of *K*_sv_ decreased with the increase of temperature, and *K*_q_ was greater than 2.0 × 10^10^ (Table 2). Therefore, the process of quenching is a static quenching by the formation of a complex.

Static quenching follows the equations [13]:

(2) $$\text{lg}\frac{F_{0}-F}{F}=\text{lg}\;K_{\text{A}}+\text{n}\;\text{lg}\;{\left[Q\right]}_{\text{f}}$$

(3) [flavonoid]f=[flavonoid]-n [α-glucosidase-flavonoid n]

(4) $$\left[\alpha -\text{glucosidase}-\text{flavonoidn}]=\frac{F_{0}-F}{F_{0}-F_{\infty }}[\alpha -\text{glucosidase}\right]$$

Here, [Q]_f_ is the concentration of free flavonoid, [flavonoid]_f_; and [α-glucosidase-flavonoid *_n_*] is the concentration of α-glucosidase bound with the flavonoid.

The vitexin binding constant (*K*_A_) is higher than the isovitexin constant (Table 3). The number of binding sites (*n*) was close to one at 37 °C, which is the most suitable temperature for the flavonoid molecules to bind with α-glucosidase.

## 3. Materials and Methods

### 3.1. Materials

Azuki beans were provided by the Chinese National Genebank (Beijing, China). Rat intestinal acetone powder was purchased from Sigma-Aldrich (St. Louis, MO, USA). Acarbose was purchased from Bayer Health Care Pharmaceuticals, Inc. (USA). All chemicals used were of analytical grade and were obtained from Beijing Chemical Reagent (Beijing, China). Silica gel (200–300 mesh) for column chromatography was purchased from Qingdao Marine Chemical Company (Qingdao, China). Sephadex LH-20 was purchased from GE Healthcare (Sweden, USA).

### 3.2. Isolation and Identification of Active Compounds

Dried Azuki beans (3.0 kg) were crushed and twice extracted with 70% ethanol (3 × 10 L) for 2 h at 60 °C. The extracts were combined and concentrated under vacuum at 50 °C. Then, the concentrated extracts were partitioned with CH_2_Cl_2_, EtOAc and n-BuOH to offer four extracts: the CH_2_Cl_2_-soluble, EtOAc-soluble, n-BuOH-soluble and residual extract fractions. Each extract was evaporated to dryness under reduced pressure, while the residual extract fraction was frozen to dryness. Therefore, five extracts were obtained in total. A small amount of each fraction was redissolved in 50% dimethyl sulfoxide (DMSO), and these mixture solutions were subjected to α-glucosidase inhibitory activity assays.

The EtOAc-soluble fraction (25 g) was subjected to a silica gel chromatography column, using an EtOAc/MeOH/H_2_O system as the eluent, and the polarity of the eluent was increased by increasing the ratio of EtOAc during the process. The separation was monitored by TLC, and four fractions were obtained. Fraction 3 [EtOAc:MeOH:H_2_O = 8:1:0.2 (v:v:v)] showed strong inhibitory activities against α-glucosidase. A further separation was completed using a combination of Sephadex LH-20 column chromatography, with MeOH as the eluent, and reversed-phase TLC to monitor the isolation.

### 3.3. Evaluation of α-Glucosidase Inhibitory Activity

The α-glucosidase inhibitory activity was determined as previously described with slight modifications [14,15]. The inhibition activity of α-glucosidase (1 unit·mL^−1^) was assayed using 50 μL of extracts with varying concentrations incubated with 100 μL of 0.1 M phosphate buffer (pH 7.0) in 96-well plates at 37 °C for 10 min. After preincubation, 50 μL of 5 mM *p*-nitrophenyl-α-dglucopyranoside solution in 0.1 M phosphate buffer (pH 7.0) was added to each well at varying time intervals. The reaction mixtures were incubated at 37 °C for 5 min. The absorbance readings were recorded at 490 nm on a microplate reader before and after incubation (BioRad, IMAX, Hercules, USA). The results were expressed as a percent of α-glucosidase inhibition and calculated according to the following equation:

(5) $$\%\;\text{inhibition}={\text{Abs}}^{\text{control}}-{\text{Abs}}^{\text{extract}}\times 100/{\text{Abs}}^{\text{control}}$$

The IC_50_ value was defined as the concentration of bean extracts (acarbose) required to inhibit 50% of the enzyme activity.

### 3.4. Measurement of Fluorescence Spectra

The fluorescence spectra were determined using the method reported by Li *et al*. [12]. The α-glucosidase was prepared by dissolving solid α-glucosidase into phosphate buffer (0.1 mol·L^−1^, pH 6.8, with 0.1 mol·L^−1^ NaCl), and vitexin (or isovitexin) was dissolved in 60% ethanol. For the FS measurement, a solution of 1.0 mL of α-glucosidase was added to a fluorescence cuvette at a given temperature and titrated with flavonoid for 5 min. Fluorescence spectra of the α-glucosidase and α-glucosidase-flavonoid mixture were recorded in the range from 315 to 500 nm. The slits for both excitation and emission were set at 10 nm with an excitation wavelength of 295 nm and an optical path of 10 mm (Hitachi F-2500 fluorescence spectrophotometer, Japan).

## 4. Conclusion

In conclusion, two major active components, vitexin and isovitexin, were isolated from the azuki bean. There is a static quenching interaction between flavonoid compounds and α-glucosidase, and the most suitable temperature is 37 °C.

## Figures and Tables

**Table 1:** Alpha-glucosidase inhibitory activity of CH_2_Cl_2_-soluble, EtOAc-soluble, *n*-BuOH-soluble, residual extract, vitexin and isovitexin.

Extracts/compounds	IC_50_
CH_2_Cl_2_-soluble	>500
EtOAc-soluble	53.74
n-BuOH-soluble,	173.69
Residual extract	>500
Vitexin	0.4
Isovitexin	4.8
Acarbose	0.45

IC_50_ was expressed as mg·mL^−1^.

**Table 2:** Constants of *K*_sv_ and *K*_q_ of the interaction between α-glucosidase and vitexin, isovitexin.

	*T* (°C)	*K*_SV_/105 (L·mol^−1^)	*K*_q_/1013 (L·mol^−1^·S^−1^)	*R*^2^
Vitexin	25	1.38	1.38	0.9436
	37	1.13	1.13	0.9627
Isovitexin	25	1.06	1.06	0.9829
	37	0.98	0.98	0.9807

**Table 3:** Values of *K*_A_ and *n* of the interaction between α-glucosidase and vitexin, isovitexin.

	*T* (°C)	*K*_A_/105 (L·mol^−1^)	*n*	*R*^2^
Vitexin	25	1.23	1.21	0.9865
	37	1.37	1.24	0.9940
Isovitexin	25	1.19	1.09	0.9671
	37	1.25	1.17	0.9513
